# The Genetic Basis of Sudden Cardiac Death: From Diagnosis to Emerging Genetic Therapies

**DOI:** 10.1146/annurev-med-042423-042903

**Published:** 2025-01-16

**Authors:** Enya R. Dewars, Andrew P. Landstrom

**Affiliations:** 1 Division of Pediatric Cardiology, Department of Pediatrics, Duke University School of Medicine, Durham, North Carolina, USA; 2 Developmental and Stem Cell Biology Program and Cell and Molecular Biology Program, Duke University School of Medicine, Durham, North Carolina, USA; 3 Department of Cell Biology, Duke University School of Medicine, Durham, North Carolina, USA

**Keywords:** sudden cardiac death, cardiomyopathy, channelopathy, genetics, gene therapy, therapeutics

## Abstract

Sudden cardiac death (SCD) is an abrupt, tragic manifestation of a number of cardiovascular diseases, primarily ion channelopathies and heritable cardiomyopathies. Because these diseases are heritable, genetics play a key role in the diagnosis and management of SCD-predisposing diseases. Historically, genetics have been used to confirm a diagnosis and identify at-risk family members, but a deeper understanding of the genetic causes of SCD could pave the way for individualized therapy, early risk detection, and a transformative shift toward genetically informed therapies. This review focuses on the evolving genetic landscape of SCD-predisposing diseases, the current state of gene therapy and therapeutic development, and the promise of using predictive genetics to identify individuals at risk of SCD.

## INTRODUCTION

Sudden cardiac death (SCD), an unexpected sudden death due to cardiovascular disease, accounts for ~50% of cardiovascular deaths worldwide and 20% of cardiovascular-related deaths in North America and Europe (1). Approximately 80% of these deaths are due to an acquired cardiovascular disease, such as ischemic heart disease, while the remaining proportion are due to heritable cardiovascular diseases (2). Among individuals under 35 years old, SCD affects 1–2 per 100,000 people; in those under 18 years, the estimated rate is 1–4 deaths per 100,000, while for young athletes, the incidence is approximately 1 in 20,000 to 50,000 (3, 4). Heritable SCD is often due to genetic variants in cardiac ion channel and channel-interacting proteins (cardiac channelopathies) or primary diseases of the myocardium (heritable cardiomyopathies) (5). As these diseases are often monogenic, the identification of the causative genetic variant is central to diagnosis, as is cascade genetic testing to identify risk in kin who may show no clinical evidence of disease (6, 7). Recent advancements in sequencing techniques, experimental models, and therapies have tremendously expanded the role of genetic testing, allowing it to inform prognosis, guide new treatments, and potentially identify disease before it occurs. In this review, we highlight the role of genetics in individualizing the management of patients with channelopathies and cardiomyopathies, driving the development of new therapies, and predicting those at risk of disease before signs or symptoms occur.

## AN EVOLVING GENETIC BASIS OF SUDDEN CARDIAC DEATH: HERITABLE CARDIOMYOPATHIES AND CARDIAC CHANNELOPATHIES

**Figure 1:**
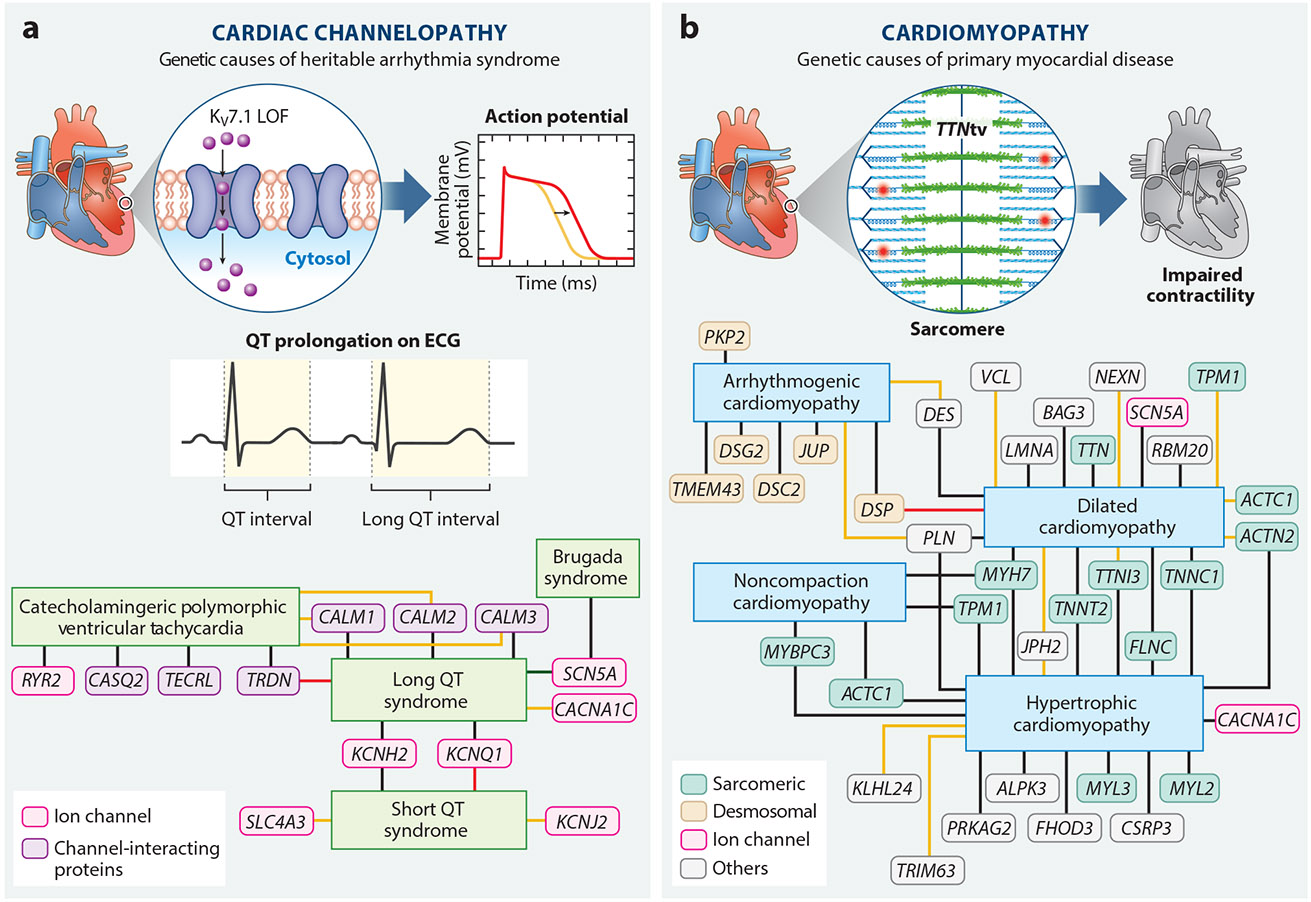
Genetic causes of sudden cardiac death–predisposing diseases. (*a, top*) The molecular basis of a common cardiac channelopathy. In this example, long QT syndrome results from a loss-of-function (LOF) mutation in *KCNQ1*, which encodes the K_V_7.1 potassium channel, leading to the prolongation of the cardiac action potential and corresponding prolongation of the QT interval on an electrocardiogram (ECG). (*a, bottom*) Genes definitively (*black line*), strongly (*red line*), and moderately (*yellow line*) associated with channelopathies based on current ClinGen assessment. (*b, top*) The molecular basis of a heritable cardiomyopathy. In this example, dilated cardiomyopathy is caused by a truncating variant in the *TTN* gene (*TTN*tv), which results in reduced contraction of the cardiac myocyte. (*b, bottom*) Genes definitively (*black line*), strongly (*red line*), and moderately (*yellow line*) associated with cardiomyopathies based on current ClinGen assessment, with the exception of genes associated with noncompaction cardiomyopathy.

The most common hereditary cardiovascular diseases that lead to SCD are cardiomyopathies and channelopathies (5) (Figure 1). With the recent expansion of genetic testing in the clinic, rare genetic variants have been newly associated with these diseases, leading to the identification of new disease states and mechanisms of disease pathogenesis. Conversely, with improved genetic tools and disease models, many genes previously associated with rare genetic causes of SCD are now being disputed. Here, we highlight genes definitively, strongly, and moderately associated with SCD (Table 1) and the shifting genetic landscape of SCD-predisposing diseases.

### Cardiomyopathies

Cardiomyopathies are primary diseases of the myocardium that develop in the absence of secondary triggers (8). Cardiomyopathies are frequent causes of SCD at any age and include hypertrophic cardiomyopathy (HCM), arrhythmogenic cardiomyopathy (ACM), nonischemic dilated cardiomyopathy (DCM), noncompaction cardiomyopathy (NCM), and restrictive cardiomyopathy (9). These cardiomyopathies are commonly associated with SCD in patients under 35 years of age (10). While the pathology of each cardiomyopathy varies, they lie along a phenotypic spectrum with a broad, overlapping genetic basis.

HCM is predominantly defined by hypertrophy of the left ventricle, with occasional involvement of the right ventricle, without an identifiable cause (11). The most common heritable cardiovascular disease, HCM affects approximately 1 per 200 to 500 individuals (12). It is often inherited in an autosomal dominant manner and is classically considered a disease of the sarcomere, the contractile unit of the cardiac myocyte. HCM typically goes underdiagnosed as patients can be asymptomatic; however, for some individuals with HCM, it is a progressive heart disease that can result in atrial fibrillation, ventricular arrhythmias, left ventricular systolic dysfunction, heart failure, and SCD (13). The most common genes associated with HCM are *MYH7*, which encodes beta myosin heavy chain, and *MYBPC3*, which encodes myosin-binding protein C3, both of which are key components of the cardiac sarcomere; other sarcomeric genes have been labeled as rare yet definitive causes of HCM, including *TNNT2*, *TNNI3*, *TPM1*, *FHOD3*, *FLNC*, *PRKAG2*, *ACTC1*, *MYL2*, *MYL3*, *TNNC1*, *CACNA1C*, *ACTN2*, and *CSRP3* (14).

ACM is a disease of the cardiac desmosome and is characterized by fibrofatty replacement of the myocardium (15). It affects approximately 1 per 2,000 to 5,000 individuals and is a leading cause of SCD in children and athletes (16, 17). Previously, ACM was known as arrhythmogenic right ventricular cardiomyopathy due to the predominant right ventricle involvement (18). Recently, left ventricle involvement has been increasingly identified as a distinct entity within ACM that is often associated with the concurrent development of inflammation and myocarditis (19, 20). A heritable disease, ACM is often seen as an autosomal dominant disorder with incomplete penetrance and variable expressivity. Approximately 60% of the genetic variants associated with ACM are found in cardiac desmosomal genes, with the most common being loss-of-function variants in *PKP2*, which encodes plakophilin 2 (PKP2), accounting for ~40% of ACM cases (21). Other ACM-associated desmosomal genes include *DSP*, *DSG2*, *DSC2*, and *JUP* (22). Autosomal recessive inheritance has also been found to be associated with desmosomal mutations, as in Naxos disease, often caused by variants in *PKP2*, and Carvajal syndrome, often caused by variants in *DSP* (23). Non-desmosomal genes that have been identified as rare yet associated with ACM include *PLN*, *DES*, and *TMEM43* (24). Recently, work by ClinGen has called into question some ACM gene–disease associations, particularly those involving rare non-desmosomal genes. For example, *LDB3* and *RYR2*, which encodes ryanodine receptor 2 (RyR2), were previously suggested to be associated with ACM development, but *LDB3*’s connection is now disputed, and *RYR2* is considered part of another genetic channelopathy (25).

DCM is defined by dilation of the left ventricle, systolic dysfunction, loss of myocytes, and myocardial fibrosis. Nonischemic DCM is not due to acquired ischemic disease but rather the disruption of a variety of cellular processes, including cellular transcription, splicing, force transduction, DNA damage, and calcium handling (26). A potentially heritable disease, DCM is autosomal dominant with more than 40 gene associations, ~20 of which encode sarcomeric and structural proteins, the most common of which is *TTN*-encoded titin (27, 28). Other genes definitively associated with DCM include *LMNA*, *TNNT2*, *RBM20*, *TNNC1*, *SCN5A*, *MYH7*, *FLNC*, *BAG3*, *PLN*, and *DES* (29). Overall, there are dozens of rare DCM-associated genes, some of which are still being discovered, highlighting the heterogeneous etiology of the disease.

Finally, NCM is a cardiomyopathy defined by excessive trabeculations resulting from disrupted compaction of the myocardium. NCM was once known as left ventricular noncompaction cardiomyopathy due to the predominant left ventricle involvement; however, both ventricles can be impacted. NCM is associated with the development of life-threatening arrhythmias, chronic heart failure, and systemic embolic events (30). In some patients with NCM, defects including congenital heart disease, neuromuscular disease, mitochondrial disease, and metabolic disease can also occur (31). Genes associated with NCM include *ACTC1*, *MYBPC3*, *MYH7*, and *TPM1* (32). As a relatively newly described cardiomyopathy, the majority of individuals with NCM do not have a diagnostic genetic variant, and a number of new candidate genes might be rare causes of cardiomyopathy. For example, disease-associated variants of *PRDM16* have recently been discovered to be associated with NCM, and loss of the *PRDM16* locus has been linked with NCM development (33-35).

### Cardiac Channelopathies

Cardiac channelopathies affect the cardiac action potential and electrical conduction system of the heart, compromising the synchronous contraction of the heart (36). Ion channelopathies include long QT syndrome (LQTS), short QT syndrome (SQTS), catecholaminergic polymorphic ventricular tachycardia (CPVT), and Brugada syndrome (BrS) (37). These diseases are often discovered through the presence of specific abnormalities on echocardiograms (ECGs) at rest or arrhythmias during exercise or adrenergic or pharmacological challenge (38).

LQTS is clinically characterized by the prolongation of cardiac repolarization time, which manifests as a prolonged QT interval on an ECG and makes the heart susceptible to ventricular arrhythmias. LQTS is one of the most common cardiac channelopathies, occurring in approximately 1 in 2,500 people, and is usually inherited as an autosomal dominant disease (37). The most commonly associated gene, accounting for ~35% of LQTS cases (i.e., type 1 LQTS), is *KCNQ1*, which encodes the K_V_7.1 voltage-dependent potassium channel. The second most common, accounting for ~30% of cases (i.e., type 2 LQTS), is *KCNH2*, which encodes K_V_11.1, the hERG (human ether-à-go-go-related gene) potassium channel. The third most common, accounting for ~10% of cases (i.e., type 3 LQTS), is *SCN5A*, which encodes the Na_V_1.5 cardiac sodium channel (37). Understanding the genetic basis of LQTS has therapeutic and prognostic implications. For example, the prevalent LQTS-associated genes *KCNQ1*, *KCNH*, and *SCN5A* have specific SCD associations, including life-threatening arrhythmias with swimming or other exercise, auditory triggers, and sleep, respectively (39). At one time, 17 genes were associated with LQTS development; however, ClinGen’s reevaluation revealed that genes such as *KCNE2*, *KCNJ5*, *SCN4B*, *SNTA1*, *AKAP9*, and *ANK2* are unlikely to be associated with LQTS (40).

SQTS is defined as abnormally fast cardiac repolarization leading to a short QT interval on an ECG. SQTS predisposes at-risk patients to supraventricular arrhythmias and SCD-associated ventricular arrhythmias (41). SQTS is an autosomal dominant disorder with nine associated genes, according to ClinGen. The three most commonly associated genes are gain-of-function mutations in *KCNH2*, *KCNQ1*, and *KCNJ2*, which encode potassium channels (42-44); however, rare variants in these genes account for ~20% of SQTS cases, so the majority of SQTS cases are genetically unexplained (45). Recently, the associations of several genes have been disputed, including *CACNA1C*, *CACNA2D1*, *CACNB2*, and *SCN54A* (46). Several emerging genes might be associated with SQTS development and are the focus of current research. For example, emerging evidence has pointed to genetic variants in *ATP1A3*, which encodes sodium/potassium ATPase 3, as a potential cause of SQTS and SCD in children with alternating hemiplegia of childhood (47).

CPVT is characterized by early-onset, high SCD-based mortality (48). CPVT manifests in supraventricular tachycardia, atrial fibrillation, ventricular extrasystoles, and bidirectional or polymorphic ventricular tachycardia, which can degenerate to ventricular fibrillation. Four genes are definitively associated with CPVT. *RYR2*, which encodes RyR2, causes the majority of CPVT cases, while *CASQ2*, which encodes calsequestrin 2, is the second most common cause of CPVT, though a rare autosomal recessive form (46, 49). CPVT-associated variants in these genes result in increased calcium leakage from the sarcoplasmic reticulum via RyR2, leading to calcium-mediated triggered arrhythmias (50, 51). *KCNJ2*, *PKP2*, and *SCN5A* are rarer causes of CPVT, but ClinGen has recently called into question their associations with CPVT development (46).

Finally, BrS is classically considered a cardiac channelopathy that commonly leads to episodes of ventricular arrhythmias. While BrS is heritable, most individuals are genotype negative (52). Approximately 18–30% of BrS cases result from variants in the *SCN5A* gene, which encodes a voltage-dependent sodium channel, leading to loss of Na_V_1.5 peak current (53). While nearly two dozen genes have been associated with BrS as rare causes, all non-*SCN5A* genes have been disputed (54). There is emerging evidence that at least some individuals with BrS who are genotypically negative for variants in *SCN5A* may have a polygenic basis for their disease (55). Further, genetic modifiers may influence the penetrance and expression of the disease (56).

## GENETICALLY INFORMED THERAPEUTICS AND GENE THERAPIES

**Figure 2:**
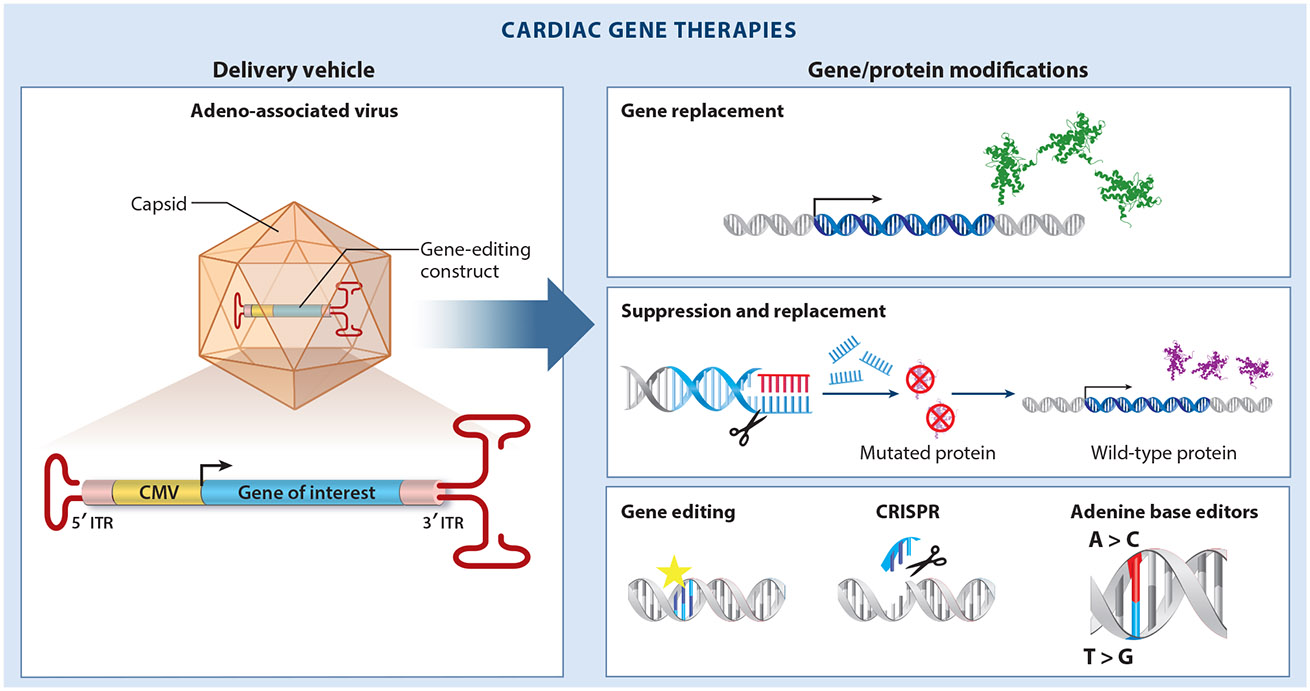
Gene therapies for cardiovascular disease. Targeted gene therapies to treat the pathogenic mechanisms of preclinical sudden cardiac death–associated disease models commonly use adeno-associated viruses to deliver genetic payloads. These payloads are targeted to the known genetic mechanism of disease pathogenesis (e.g., gene replacement for loss-of-function variants; suppression of disease-associated alleles and replacement with a normally functioning gene for dominant-negative mechanisms; DNA editing through genetic deletion, correction of disease-associated variants, or replacement of single nucleotides). Abbreviations: CMV, cytomegalovirus; CRISPR, clustered regularly interspaced short palindromic repeats; ITR, inverted terminal repeats.

The current state of therapeutics for SCD-predisposing diseases is rapidly evolving. Such therapeutics were once limited to pharmacotherapy, device-based therapy, and ablative therapy, but genetics has catalyzed a new era of therapeutic discovery, including small molecules informed by genetic mechanisms of disease as well as gene therapies (Figure 2). These genomic-era approaches have the potential to transform the care landscape for patients.

### Small Molecules Targeting Sarcomeric Proteins

Advanced HCM can be challenging to treat, with many patients experiencing symptoms and mortality despite traditional therapies. The US Food and Drug Administration recently approved mavacamten, a small-molecule, cardiac-specific myosin inhibitor used to treat obstructive HCM (57). This small-molecule inhibitor is the first genetic mechanism and sarcomeric protein–specific treatment for HCM in patients with refractory symptoms and disease. Mavacamten targets the core mechanism of HCM. In preclinical work, many sarcomeric HCM variants, such as those in *MYH7* and *MYPBC3*, cause an increased proportion of myosin heads to be in a disordered relaxed state, disrupting the balance between this state and a super-relaxed state. This increases the number of actin interactions, resulting in a hypercontractile state (58, 59). When applied to HCM mouse models carrying human heterozygous mutations in *Myh7* (i.e., MHC^403/+^, MHC^719/+^, and MHC^453/+^), mavacamten normalized the hypercontractile myosin heavy chain, suppressed ventricular hypertrophy, and improved myocardial fibrosis (60). Success with mavacamten has led to the creation of similar molecules, which are currently in development. For example, aficamten, another myosin inhibitor, is being evaluated for HCM treatment (61). Parallel efforts are underway to identify similar precision medicine therapies for other cardiomyopathies. For example, danicamtiv, a myosin activator, is currently in phase II clinical trials as a treatment method for systolic heart failure and DCM. Danicamtiv has been shown to increase force and calcium sensitivity in cardiac muscle by increasing myosin recruitment and altering the cross-bridge cycle (62). Preclinical studies have been exploring the use of other myosin activators (e.g., omecamtiv) to treat cases of DCM where the defect is in loss of myosin contractility (63). These advances, which leverage the genetic mechanisms of disease to inform therapeutic interventions, represent a transformative aspect of genomic medicine for cardiovascular disease (64).

### Gene Therapy for Cardiomyopathies and Channelopathies

Within the last three decades, gene therapy has shown promise for treating many heritable diseases, including SCD-associated diseases. The cardiotropic adeno-associated virus (AAV) serotype 9 has become a tractable model for its ease of use, nonintegrating delivery, cardiac specificity, and theoretically low immune response in humans.

#### Gene replacement.

Cardiomyopathies have been a major beneficiary of gene replacement strategies, particularly in disease states marked by loss-of-function mutations and a haploinsufficient genetic mechanism. For example, given that *PKP2* loss-of-function mutations are the most common cause of ACM, AAV9–*PKP2* overexpression has been pursued as an approach to treating the disease. AAV9–*PKP2* treatment led to restoration of the disrupted desmosomal complex, prevented cardiac dysfunction, and improved contractile function in a heterozygous *Pkp2*^c.1755delA^ knock-in mouse model without adversely affecting the wild-type (WT) control mice. Similar results were found in a patient-derived *PKP2*^c.2013delC/WT^ induced pluripotent stem cell cardiac myocyte (iPSC-CM) line compared to the WT (65). This approach has also been successful in *LAMP2* (lysosomal-associated membrane protein 2) replacement therapy for Danon disease. Specifically, *Lamp2* knockout mice treated with AAV9–*LAMP2B* had a dose-dependent increase in LAMP2B protein expression in the heart, liver, and skeletal muscle. Mice treated with AAV9–*LAMP2B* had improved cardiac function, improved metabolic function, and reduced liver function impairment (66). Finally, 1-day-old homozygous *Mybpc3*-targeted knock-in mice treated with AAV9–*Mybpc3* demonstrated increased total protein levels of myosin binding protein C (MyBP-C) and prevention of cardiac hypertrophy development (67). This preclinical work has directly translated to early-stage clinical trials for each of these gene therapy approaches. While these strategies hold tremendous promise, they come on the heels of the CUPID Trial, which demonstrated no efficacy. The CUPID Trial was a phase II trial attempting to treat ischemic DCM with AAV1 gene transfer of sarco/endoplasmic reticulum calcium ATPase 2a (SERCA2a). Loss of SERCA2a is a consistent finding in the molecular remodeling of ischemic heart failure and DCM (68); however, AAV1–SERCA2a coronary infusion did not improve cardiac outcomes (69). It is unclear why no efficacy was observed. Innovative modified replacement strategies have also been pursued for molecular targets that are too large to fit into traditional AAV capsids. Human *SCN5A*, which encodes the Na_V_1.5 cardiac sodium channel, is ~80 kb, which is too large to fit into an AAV for direct replacement in patients with BrS. Recently, a unique gene therapy leveraging BacNa_V_, a much smaller, engineered prokaryotic sodium channel, was developed (70). When this prokaryotic sodium channel was expressed through self-complementary AAV9, it improved excitability and action potential conduction in vitro in rat and human cardiac myocytes. Together, these examples highlight the leading edge of gene therapy approaches for SCD-associated cardiovascular disease.

#### Suppression and replacement, indirect modulators, and molecular chaperones.

While gene replacement has been pursued for loss-of-function, haploinsufficient mutations, a suppression- and-replacement (SupRep) method has been proposed for reducing the expression of a mutant allele while simultaneously normalizing the expression of the WT allele. The first proof-of-concept hybrid gene therapy model was demonstrated for *KCNQ1-* and *KCNH2*-mediated LQTS and SQTS using short hairpin RNAs in a dual-component SupRep method, which silenced the mutant allele and replaced it with WT expression (71, 72). SupRep was shown to silence the disease-associated variant while simultaneously replacing its expression with that of a modified gene of interest using a short hairpin RNA-immune construct in vitro. This resulted in the normalization of action potential duration in iPSC-CMs. In vivo studies testing SupRep in LQTS are currently underway. In addition to replacing lost gene products, there have been advances in targeting specific signaling pathways that promote cardiovascular disease states. For example, calcium/calmodulin-dependent kinase II (CaMKII), a calcium-sensing kinase regulator, contributes to arrhythmias and has been considered a prime therapeutic target for CPVT (73). Using an Ryr2^R176Q/+^ mouse model of CPVT and two iPSC-CMs derived from CPVT patients, AAV9-mediated delivery of a CaMKII inhibitory peptide, autocamtide-2-related inhibitory peptide, was shown to suppress triggered arrhythmias (74). Similarly, in a heterozygous *Scn5a*^G1746R/+^ BrS knock-in mouse model, overexpression of the multicopy suppressor of Gsp1 (MOG1) chaperone protein, which mediates proper trafficking of Na_V_1.5 to the cell surface, was found to increase cell surface expression of Na_V_1.5, normalizing cardiac action potential abnormalities, cardiac conduction, and arrhythmias (75). These approaches have shown tremendous promise in treating complex genetic mechanisms of disease when traditional gene replacement is not feasible.

#### Base editing.

Programmable base editing, such as using clustered regularly interspaced short palindromic repeats (CRISPR) to alter adenine bases by converting A and T base pairs to C and G, has enabled the permanent modification of genetic variants (76, 77). Recently, a LQTS mouse model with an Scn5a-T1307M pathogenic variant was treated with AAV9 containing the adenine base editor to correct the *Scn5a* mutant transcripts. Up to 99.20% of the *Scn5a* transcripts were corrected, resulting in an improvement in the arrhythmic phenotype seen in this model before treatment (78). Similar approaches have been successful in treating other cardiovascular diseases. To reduce hepatic production of cholesterol in patients with heterozygous familial hypercholesterolemia and atherosclerotic cardiovascular disease, CRISPR base editors were used to change a single DNA nucleotide in proprotein convertase subtilisin/kexin type 9 (*PCSK9*) to reduce its function. Base editing in primates led to a durable lowering of PCSK9 in the liver and a subsequent drop in low-density lipoprotein cholesterol (79).

#### Targeted payload delivery.

A limitation in the field of cardiovascular gene therapy is the challenge of organ-specific delivery of gene therapy payloads (80). While AAV9 is the most cardiotropic viral vector for cardiovascular delivery, improved specificity of gene delivery to the heart is still needed, as high levels of viral expression continue to be observed in the liver (81). Studies have demonstrated ways to circumvent this challenge by altering the viral capsid. Cardiotropic capsids, such as the chimeras AAV.cc47, AAV2i8G9, and AAV-SASTG, have demonstrated higher cargo delivery specifically to the heart (82, 83). As a result, a cardiotropic capsid, BNP116—an AAV2/AAV8 chimera—is currently undergoing clinical trials for heart failure. A phase I dose escalation trial has begun using AAVrh.74 containing human *PKP2* coding sequences to address patients with ACM with a pathogenic *PKP2* variant (84). Similarly, a different group is currently conducting early-phase clinical trials using AAVrh.10h encoding the *PKP2* gene to study the safety and efficacy of gene therapy in patients with PKP2-mediated ACM (85).

## THE EMERGENCE OF PREDICTIVE CARDIOVASCULAR GENETICS

**Figure 3:**
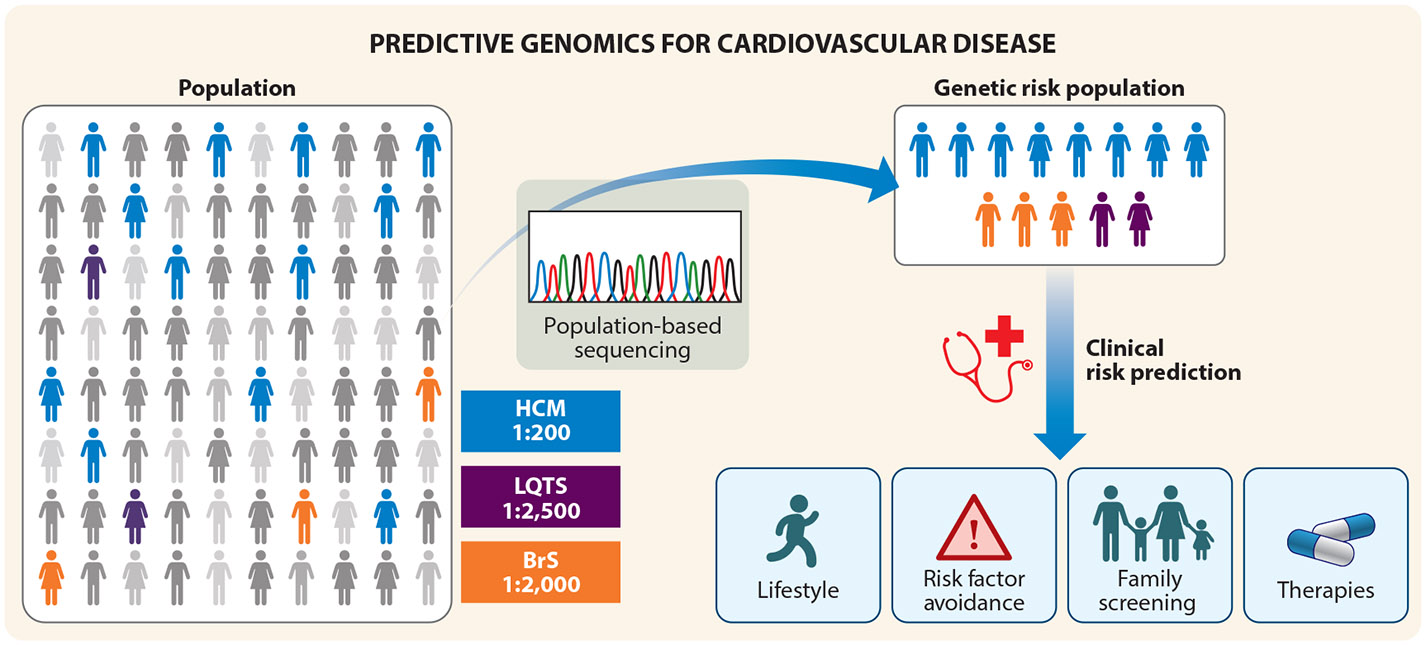
Predictive genomics for the early detection and mitigation of SCD-predisposing disease risk. Genomic screening of a population may identify those at risk of developing SCD-predisposing disease, regardless of symptoms. Individuals found to be at genetic risk can then be assessed by a comprehensive and multidisciplinary clinical evaluation that is individualized to the person and the variant identified. Those found to be at clinical risk may then be offered a suite of interventions, such as lifestyle modifications, risk factor avoidance, and interventional cardiovascular therapies, when appropriate, to lower the risk of mortality. Abbreviations: BrS, Brugada syndrome; HCM, hypertrophic cardiomyopathy; LQTS, long QT syndrome; SCD, sudden cardiac death.

With the increasing ease and accessibility of broad genetic testing, identifying heritable disease early in life through predictive genetic testing has become an emerging possibility. Individuals often do not have any symptoms until SCD occurs; however, predictive genetic testing could potentially be used to identify those with SCD-associated genetic variants before symptoms appear (Figure 3). The plummeting cost of genome sequencing and the widespread availability of clinical exome and genome sequencing has already enabled predictive genetic testing among research cohorts, such as those in the United Kingdom (86, 87). Despite the tremendous promise, a number of barriers stand in the way of successful implementation. The American College of Medical Genetics (ACMG) has established guidelines to report incidental findings, also known as secondary findings, in specific genes that are clinically actionable (88). Further, there are ACMG guidelines for interpreting variants to establish the probability that they are associated with disease (89). This provides a valuable foundation for predicting whether or not the genetic variants cause disease. Nevertheless, it is becoming increasingly recognized that population-based sequencing will reveal more presumptive disease-associated variants than the actual prevalence of the disease in the population (90). This suggests either the identification of low penetrance alleles or perhaps inappropriately designated disease associations. Recent advances in artificial intelligence, population genomic modeling, and high-throughput functional platforms could potentially be used to accurately predict which variants are most likely to result in penetrant disease (91-94). Validation of these tools in large-scale genome and phenome biobank datasets (e.g., *All of Us*, UK BioBank, BioVU at Vanderbilt, Helix Research Network) is critical to determine whether predictive genetic testing can be done for SCD-predisposing diseases at a population scale. For example, recent work with the UK BioBank showed that putatively pathogenic variants in DCM were likely low penetrance alleles, yet they influenced cardiovascular mortality (95, 96). This highlights the need to evaluate variants for SCD-predisposing disease genes on an individual basis, taking into account the specific variant, the associated gene, and the individual who carries the variant (97). Accurate interpretation of genetic variants relies on a thorough understanding of the complex genetic diversity present worldwide (98). In addition to the complexities of genetic testing, it is important to consider legislative protections against genetic discrimination, which vary by region; privacy concerns surrounding the genetic testing of an individual; and the potential implications for family members (99).

## ON THE HORIZON

Modern genomics holds seemingly endless opportunities to improve human health and individualize therapeutic approaches. A critical future direction is to diversify population genomics studies to include underrepresented communities. Expanding what is known about the spectrum of variants across diverse ancestries is central to predicting which variants are likely to cause disease and which are not. Additionally, further work should emphasize the ethical, legal, and social implications of genomic sequencing for patients and patient groups. This will help support the use of large-scale early genomic sequencing in young individuals and enhance its role in predictive medicine. Alongside advancements in genomic sequencing, there are increasing opportunities to study more pathogenic variants in preclinical models. For example, techniques such as genome saturation mutagenesis combined with high-throughput functional analyses can be used to comprehensively assess how genetic variation affects the encoded protein. Lastly, efforts can be expanded to further emphasize the importance of equitable and diverse patient enrollment in clinical trials to ensure the discovery of a therapy that may benefit more people. Overall, the ever-growing field of cardiovascular medicine is increasingly focusing on the power of genetic studies and individualized therapies.

## Figures and Tables

**Table 1:** Genes and corresponding proteins associated with cardiac channelopathies and cardiomyopathies

Disease	Gene	Locus	Protein	Classification^a^	Frequency
Cardiac channelopathies					
Long QT syndrome	*KCNQ1*	11p15.5	Potassium voltage-gated channel subfamily Q member 1 (K_V_7.1)	Definitive	35%
	*KCNH2*	7q36.1	Potassium voltage-gated channel subfamily H member 2 (K_V_11.1)	Definitive	30%
	*SCN5A*	3p22.2	Sodium voltage-gated channel alpha subunit 5 (Na_V_1.5)	Definitive	10%
	*CALM1*	14q32.11	Calmodulin 1	Definitive	Rare
	*CALM2*	2p21	Calmodulin 2	Definitive	Rare
	*CALM3*	19q13.32	Calmodulin 3	Definitive	Rare
	*TRDN*	6q22.31	Triadin	Strong	Rare
	*CACNA1C*	12p13.33	Calcium voltage-gated channel subunit alpha1 C	Moderate	Rare
Catecholaminergic polymorphic ventricular tachycardia	*RYR2*	1q43	Ryanodine receptor 2	Definitive	50–60%
	*CASQ2*	1p13.1	Calsequestrin 2	Definitive	2–5%
	*TECRL*	4q13.1	Trans-2,3-enoyl-CoA reductase-like protein	Definitive	Rare
	*TRDN*	6q22.31	Triadin	Definitive	Rare
	*CALM1*	14q32.11	Calmodulin 1	Moderate	Rare
	*CALM2*	2p21	Calmodulin 2	Moderate	Rare
	*CALM3*	19q13.32	Calmodulin 3	Moderate	Rare
Brugada syndrome	*SCN5A*	3p22.2	Sodium voltage-gated channel alpha subunit 5 (Na_V_1.5)	Definitive	18–30%
Short QT syndrome	*KCNH2*	7q36.1	Potassium voltage-gated channel subfamily H member 2 (K_V_11.1)	Definitive	15%
	*KCNQ1*	11p15.5	Potassium voltage-gated channel subfamily Q member 1 (K_V_7.1)	Strong	11%
	*KCNJ2*	17q24.3	Potassium inwardly rectifying channel subfamily J member 2	Moderate	<1%
	*SLC4A3*	2q35	Solute carrier family 4 member 3	Moderate	Rare
Heritable cardiomyopathies					
Hypertrophic cardiomyopathy	*MYBPC3*	11p11.2	Myosin-binding protein C3	Definitive	25–35%
	*MYH7*	14q11.2	Beta myosin heavy chain	Definitive	25–35%
	*TNNT2*	1q32.1	Troponin T, cardiac type	Definitive	3–5%
	*TNNI3*	19q13.42	Troponin I, cardiac type	Definitive	1–5%
	*TPM1*	15q22.2	Alpha tropomyosin	Definitive	1–5%
	*FHOD3*	18q12.2	Formin homology 2 domain containing 3	Definitive	2%
	*FLNC*	7q32.1	Filamin C	Definitive	1–2%
	*ALPK3*	15q25.3	Alpha kinase 3	Definitive	1–2%
	*PRKAG2*	7q36.1	Protein kinase AMP-activated non-catalytic subunit gamma 2	Definitive	<1%
	*JPH2*	20q13.12	Junctophilin 2	Moderate	<1%
	*ACTC1*	15q14	Alpha cardiac actin	Definitive	Rare
	*MYL2*	12q24.11	Regulatory myosin light chain	Definitive	Rare
	*MYL3*	3p21.31	Essential myosin light chain	Definitive	Rare
	*TNNC1*	3p21.1	Troponin C, cardiac type	Definitive	Rare
	*CACNA1C*	12p13.33	Calcium voltage-gated channel subunit alpha1 C	Definitive	Rare
	*ACTN2*	1q43	Actinin alpha 2	Definitive	Rare
	*CSRP3*	11p15.1	Cysteine- and glycine-rich protein 3	Definitive	Rare
	*KLHL24*	3q27.1	Kelch-like family member 24	Moderate	Rare
	*TRIM63*	1p36.11	Tripartite motif containing 63	Moderate	Rare
Dilated cardiomyopathy	*TTN*	2q31.2	Titin	Definitive	15–20%
	*LMNA*	1q22	Lamin A/C	Definitive	5–13%
	*TNNT2*	1q32.1	Troponin T, cardiac type	Definitive	3–6%
	*RBM20*	10q25.2	RNA-binding motif protein 20	Definitive	2–6%
	*MYH7*	14q11.2	Beta myosin heavy chain	Definitive	1–5%
	*DSP*	6p24.3	Desmoplakin	Strong	3%
	*SCN5A*	3p22.2	Sodium voltage-gated channel alpha subunit 5 (Na_V_1.5)	Definitive	2–3%
	*BAG3*	10q26.11	BAG cochaperone 3	Definitive	2–3%
	*FLNC*	7q32.1	Filamin C	Definitive	1–4%
	*DES*	2q35	Desmin	Definitive	1–2%
	*TPM1*	15q22.2	Tropomyosin 1	Moderate	1–2%
	*ACTC1*	15q14	Alpha cardiac actin	Moderate	<1%
	*NEXN*	1p31.1	Nexilin F-actin binding protein	Moderate	<1%
	*VCL*	10q22.2	Vinculin	Moderate	<1%
	*PLN*	6q22.31	Phospholamban	Definitive	Rare
	*TNNC1*	3p21.1	Troponin C, cardiac type	Definitive	Rare
	*ACTN2*	1q43	Actinin alpha 2	Moderate	Rare
	*JPH2*	20q13.12	Junctophilin 2	Moderate	Rare
	*TNNI3*	19q13.42	Troponin I3, cardiac type	Moderate	Rare
Arrhythmogenic cardiomyopathy	*PKP2*	12p11.21	Plakophilin 2	Definitive	10–45%
	*DSP*	6p24.3	Desmoplakin	Definitive	10–20%
	*DSG2*	18q12.1	Desmoglein 2	Definitive	10%
	*DSC2*	18q12.1	Desmocollin 2	Definitive	Rare
	*JUP*	17q21.2	Plakoglobin	Definitive	Rare
	*TMEM43*	3p25.1	Transmembrane protein 43	Definitive	Rare
	*DES*	2q35	Desmin	Moderate	Rare
	*PLN*	6q22.31	Phospholamban	Moderate	Rare
Noncompaction cardiomyopathy	*ACTC1*	15q14	Alpha cardiac actin	NA	Rare
	*MYBPC3*	11p11.2	Myosin-binding protein C3	NA	Rare
	*MYH7*	14q11.2	Beta myosin heavy chain	NA	Rare
	*TPM1*	15q22.2	Alpha tropomyosin	NA	Rare

Abbreviation: NA, not assessed by ClinGen as of May 2024.

a Classification of gene–disease relationship by ClinGen as of May 2024.

## SUMMARY POINTS

1. The genomic landscape of sudden cardiac death (SCD)–associated disease is shifting. Several genes previously associated with cardiomyopathies and channelopathies have been disputed due to the lack of rigorous evidence.
2. Large-scale genomic sequencing has the potential to transform disease management into a more personalized form of preventative cardiovascular care.
3. Preclinical models have yielded promising results for several gene therapeutic strategies targeting SCD-associated genetic disease.
4. Predictive genetics could potentially be used to identify those at risk of SCD-predisposing diseases before signs or symptoms develop.
5. Genetic diversity in sequencing enhances the ability to accurately identify true pathogenic variants within specific populations.

## Abbreviations

SCD: sudden cardiac death
HCM: hypertrophic cardiomyopathy
ACM: arrhythmogenic cardiomyopathy
DCM: dilated cardiomyopathy
NCM: noncompaction cardiomyopathy
LQTS: long QT syndrome
SQTS: short QT syndrome
CPVT: catecholaminergic polymorphic ventricular tachycardia
BrS: Brugada syndrome
AAV: adeno-associated virus
iPSC-CM: induced pluripotent stem cell cardiac myocyte
